# Clinical Indications and Utilisation Patterns of Cardiac MRI in a Rural Hospital: Highlighting the Clinical Impact and Significance of the Service Delivery Model

**DOI:** 10.7759/cureus.114960

**Published:** 2026-08-22

**Authors:** Arslan K Raja, Nain Tara Raja, Sara Raja, John Tracey, Jonathan P Ashmore, Chetan Morey, Alex SC Ching

**Affiliations:** 1 Department of Radiology, University of Edinburgh, Edinburgh, GBR; 2 Department of Radiology, School of Medicine and Dentistry, University of Aberdeen, Aberdeen, GBR; 3 Department of Medical Physics, Raigmore Hospital, NHS Highland, Inverness, GBR; 4 Department of Medical Physics and Bioengineering, Raigmore Hospital, NHS Highland, Inverness, GBR; 5 Department of Radiology, Raigmore Hospital, NHS Highland, Inverness, GBR

**Keywords:** cardiac magnetic resonance, cardiomyopathy, diagnostic yield, gadolinium contrast, myocarditis, rural health services

## Abstract

Aim/objective: The aim/objective of the study is to evaluate the clinical indications, utilisation patterns, technical performance, and diagnostic impact of cardiac magnetic resonance imaging (CMR) in a rural general hospital between 2024 and 2025.

Materials and methods: This retrospective observational study was conducted in a rural general hospital where CMR was used as part of routine clinical services. All patients undergoing CMR between November 1, 2024, and October 30, 2025, were included (n = 114). Data collected included patient demographics, clinical indications, study type, contrast use, image quality, diagnostic findings, and clinical impact.

Results: Of 114 CMR scans, 83 (72.8%) were outpatient studies, with a mean patient age of 52.97 years (approximately 53 years). The most common indications were cardiomyopathy assessment, accounting for 21 (18.4%) cases; myocarditis evaluation, 15 (13.2%) cases; and ischaemia assessment, 12 (10.5%) cases. Furthermore, 111 (97.4%) scans used gadolinium contrast. Image quality was satisfactory or good in 92 (80.7%) of examinations. Myocardial scar or fibrosis was the most common finding, seen in 51 (44.7%) cases, followed by myocarditis, found in 24 (21.1%) cases. CMR provides diagnostic confirmation or exclusion in 46 (40.4%) cases, identifies a new diagnosis in 28 (24.6%) cases, and influences management in 19 (16.7%) cases. No major complications were documented; minor tolerability issues, including breath-holding difficulty, occurred in six (5.3%) examinations.

Conclusions: CMR is a valuable diagnostic tool in rural settings, providing high diagnostic yield and influencing clinical management despite service limitations. These findings highlight the importance of sustaining and expanding rural CMR services to reduce diagnostic inequality.

## Introduction

Cardiovascular disease (CVD) remains a leading cause of mortality and disability worldwide, placing significant pressure on healthcare systems to deliver timely and accurate diagnostic services. As the prevalence of hypertension, obesity, and diabetes increases, the diagnostic demands placed on cardiac services also continue to increase [1]. This escalation highlights the need for accurate imaging modalities that provide a comprehensive assessment of cardiac structure and function [2]. The literature has shown that cardiac magnetic resonance imaging (CMR) has emerged as a leading solution to this, providing detailed tissue characteristics beyond other imaging methods [2,3].

Unlike other imaging methods, such as computed tomography (CT) and echocardiography, CMR has been designed to provide a comprehensive evaluation of ventricular function, viability assessment, fibrosis quantification, myocardial perfusion, and other tissue characteristics within a single examination [4]. Therefore, due to its reliable results, it is considered the preferred gold standard imaging technique to assess cardiac volumes and ejection fractions [5]. An important component of CMR is late gadolinium enhancement (LGE) [6], which uses a gadolinium-based contrast agent to effectively visualise any areas of scar or fibrosis on the myocardium, which is an essential indicator for conditions such as ischaemic heart disease, myocarditis, and cardiomyopathies [7].

Evidence specifically examining CMR utilisation outside tertiary centres remains limited. Published studies from district general hospitals have demonstrated that CMR can be incorporated into routine clinical practice outside major tertiary centres, with assessment of clinical indications, image quality, diagnostic findings, and impact on patient management. However, there is comparatively little evidence describing CMR utilisation within rural general hospitals and the characteristics of locally delivered rural CMR services. In particular, limited data are available regarding referral indications, types of CMR examinations performed, protocol availability, technical image quality, diagnostic findings, and documented clinical impact within these settings [7,8].

In this study, the term ‘rural’ refers specifically to a general hospital serving a predominantly rural population within a high-income healthcare system. The rural setting described in this study therefore differs from rural healthcare environments in low- and middle-income countries, where differences in infrastructure, workforce, funding, and access to advanced imaging may be substantially greater. Accordingly, the findings of this study should be interpreted within the context of the local healthcare system and should not be assumed to be directly generalisable to rural settings internationally.

This represents an important knowledge gap because the availability of a CMR service does not necessarily reflect how the service is utilised or the clinical role it provides. Our study therefore provides real-world service-level data from a rural general hospital over 12 months, characterising CMR indications, utilisation patterns, technical performance, diagnostic findings, and documented clinical impact. This may provide useful evidence for understanding the feasibility and role of locally delivered CMR services outside major tertiary centres.

Recent guideline updates released between 2024 and 2025 by international bodies such as the European Society of Cardiology (ESC), American Heart Association (AHA), and Society for Cardiovascular Magnetic Resonance (SCMR) have expanded the clinical indications for CMR [8]. These indications include its use in evaluating heart failure, chest pain pathways, arrhythmia, cardiomyopathy, and pre-procedural planning. Due to its increased demand, the appropriate utilisation of CMR has become extremely crucial for providing effective patient care.

However, despite its usefulness in clinical practice, the accessibility of CMR remains uneven across different healthcare settings. For example, research has shown that rural hospitals often face financial and workforce challenges that affect their ability to use advanced diagnostic services [9,10]. These may be explained by limited MRI scanner time allocation, a decreased number of trained cardiologists or radiologists, or restricted funding available [10]. Also, patients living in rural settings often have delayed clinical diagnosis due to long travel distances or interrupted continuity of care [10]. Furthermore, research has shown that the rural population often experiences a higher prevalence of CVD yet has lower access to specialist diagnostic methods such as CMR [11].

The imbalances raise important questions about how CMR is utilised in rural settings, because there is a resource-demand mismatch in many rural hospitals, and a more robust, efficient and effective service model catering to the escalating clinical demands in cardiovascular imaging is required. For example, factors affecting this decision may include clinicians’ familiarity with the guidelines, local referral pathways, or clinical confidence upon acting on results. Understanding this is important to prevent inequalities in care provided to patients. If CMR is underutilised, then certain diseases, such as cardiomyopathies, arrhythmias, or myocarditis, may be missed or underdiagnosed [12].

Despite its recognised value, little is known about how CMR is utilised in rural general hospitals. This study therefore aims to evaluate CMR activity in a rural general hospital during 2024-2025, examining patient characteristics, referral patterns, clinical indications, utilisation behaviours, and clinical diagnostic impact.

## Materials and methods

The project aims to better understand how CMRs are used in a rural environment. To achieve this, all CMRs performed during this time period were analysed to assess patient characteristics, clinical indications, and diagnostic outcomes.

CMR examinations were performed at the rural general hospital using two 1.5 T MRI scanners manufactured by General Electric Healthcare (Chicago, IL, USA). A standardised CMR protocol with an approximate acquisition time of 45 minutes was used. Following patient positioning, localiser images were acquired to facilitate cardiac plane prescription, followed by standard cardiac imaging planes including two-chamber, four-chamber, three-chamber, left ventricular (LV) outflow tract, and short-axis cine imaging.

The four clinical-impact outcomes were treated as mutually exclusive categories, with each CMR examination assigned a single primary outcome. Where more than one outcome was potentially applicable, scans were classified according to the following hierarchy: diagnostic confirmation/exclusion, new/unexpected diagnosis, aid to patient management, and reassurance.

This retrospective observational study was conducted at a rural general hospital serving a predominantly rural population within the UK healthcare system. The hospital provides access to advanced diagnostic imaging, including CMR, within an established healthcare infrastructure. The term ‘rural’ in this study refers to the geographical and population characteristics of the hospital’s catchment area and does not imply limited healthcare infrastructure comparable to rural settings in low- or middle-income countries.

Gadolinium-based contrast was administered in 111 (97.4%) examinations. Contrast was administered using an automated injector from the CMR control room, followed by LGE imaging. Three examinations were performed without contrast: two due to pregnancy and one due to a documented prior contrast allergy. Pharmacological stress CMR was not performed during the study period.

Data collection involved using an Excel file (Microsoft Corp., Redmond, WA, USA) exported from the hospital’s Radiology Information System (RIS) and Picture Archiving and Communication System (PACS). This dataset provided an overview of each imaging study and included information about the site where the scan was performed (e.g., chest), whether the exam was outsourced, imaging modality, patient identification numbers, study status, order status, number of images acquired, a brief study description, accession number, patient type (inpatient vs outpatient), referring clinician, priority status (non-urgent vs urgent), whether the reports had an addendum, details of the clinician who drafted and signed the report, RIS, and PACS codes. These fields were used to identify all CMR cases and to confirm that each case had a complete imaging report available for review.

Clinical impact was defined as the documented consequence of the CMR examination for diagnostic assessment. The clinical impact of each examination was retrospectively categorised into one of four mutually exclusive primary outcomes: (1) diagnostic confirmation or exclusion, where the CMR confirmed or excluded the suspected diagnosis or clinical concern prompting referral; (2) new or previously unrecognised diagnosis, where the CMR identified a clinically relevant abnormality or diagnosis that had not been established before imaging; (3) change or direct influence on patient management, where the CMR findings resulted in a documented change in treatment, further investigation, referral, or clinical management; or (4) reassurance, where no significant abnormality was identified and the CMR provided diagnostic reassurance without a documented change in management.

After the initial stage, the second stage of data extraction involved obtaining the clinical details needed for the study. This involved using written, finalised CMR reports to extract different clinical variables, such as the main indication for the CMR, such as assessment for cardiomyopathy, ischaemic evaluation, myocarditis, or other clinical concerns.

Parametric mapping techniques, including native T1/T2 mapping and extracellular volume (ECV) quantification, were not routinely performed during the study period. Tissue characterisation primarily relied on conventional sequences and LGE.

Other data collected involved examining whether the CMR led to any change in patient management. This included documenting whether the scan resulted in a new unexpected diagnosis, helped to confirm a diagnosis, aided in patient management, or reassured patients. Furthermore, technical details of the scan were extracted, including the type of CMR performed (non-stress, adenosine stress, or dobutamine stress), the image quality (optimal, satisfactory, or suboptimal), whether contrast was used, and the specific contrast agent administered. Image quality was categorised as satisfactory when the examination was considered diagnostically adequate and substandard when technical limitations, such as motion artefact, poor breath-holding, or arrhythmia-related artefact, reduced diagnostic image quality. All of these variables were included in the original Excel file to create a complete file containing both administrative and clinical information.

Once all the data had been entered, the reports were then checked for any missing values, inconsistencies, and duplicate records. Moreover, to ensure that confidentiality is maintained, any patient-identifiable information was removed. Data analysis included both descriptive and statistical inference. Percentages and frequencies were used to summarise categorical variables. Chi-squared goodness-of-fit tests were performed to see whether observed distributions of categorical variables were different from an equal expected distribution. Pearson residuals were analysed to determine which categories contributed to the observed differences. Wilson 95% confidence intervals (CIs) were calculated for selected proportions. A p-value < 0.05 was considered statistically significant.

Clinical indications, principal CMR findings, and clinical-impact outcomes were categorised as mutually exclusive variables, with each examination assigned a single primary category. Continuous variables, such as the patient's age, were summarised using means, whereas categorical data, such as clinical indication, referral source, and diagnostic findings, were shown as percentages. The purpose of the analysis was to focus on identifying patterns in utilisation, the most common indications for CMR, the percentage of studies performed with contrast, and the prevalence of key diagnostic features.

Myocarditis/inflammatory changes were defined according to established CMR features, including myocardial oedema and non-ischaemic myocardial injury on LGE, in accordance with the Lake Louise Criteria. Cardiomyopathy/LV dysfunction was defined based on documented structural or functional abnormalities, including reduced LV systolic function, ventricular dilatation or hypertrophy, and characteristic myocardial tissue abnormalities reported on CMR.

Moreover, representative CMR images included in this study were taken from patients included within the study cohort. These images were used to show common diagnostic findings during the study period.

## Results

This retrospective observational study included 114 CMR scans performed between November 1, 2024, and October 30, 2025, at a rural general hospital. This group included a diverse population of both hospital inpatients and outpatients; however, approximately 83 (72.8%) of these scans occurred in an ambulatory care setting and were therefore primarily electively used.

The age range of this patient group was 18 to 85 years, with an average age of 52.97 ± 2.88 years. The age range was 18-85 years, reflecting the broad age range of patients referred for CMR during the study period.

In this group, the results of this report suggest a greater proportion of men than women (n = 69, 60.5% male). The higher proportion of men than women in this study population is consistent with the established demographic patterns of many of the conditions that are the primary reasons patients undergo CMR. These conditions include cardiomyopathy, myocarditis, and ischaemic heart disease. Baseline characteristics are shown in Table 1. CMR was ordered by a wide range of medical professionals from different disciplines of care (most commonly cardiology, general medicine, and acute medical teams).

**Table 1:** Patient Characteristics of the Cardiac MRI Cohort Baseline characteristics of the cardiac MRI cohort (n = 114). Continuous variables are shown as mean and range. Categorical variables are shown as number and percentage with 95% confidence intervals. Chi-squared goodness-of-fit tests were used to show categorical distributions against an equal expected distribution. χ²: chi-squared statistic

Demographic characteristic	Value	95% confidence interval	χ² statistic	p-value
Age, mean (years)	52.97 ± 2.88	-	-	-
Age range (years)	18-85	-	-	-
Male, n (%)	69 (60.5%)	51.4-69.0		
Female, n (%)	45 (39.5%)	31.0-48.7		
Inpatient, n (%)	31 (27.2%)	19.9-36.0	χ² = 23.72	<0.001
Outpatient, n (%)	83 (72.8%)	64.0-80.1		

In this study, we analysed the use of CMR across a range of clinical settings to improve clarity and ensure consistent reporting in line with existing frameworks recommended by the ESC and the SCMR. The results of this study highlighted that the top two indications for CMR were evaluation of cardiomyopathy (n = 21, 18.4%), including suspected or diagnosed cardiomyopathies requiring additional characterisation of phenotype, evaluation of left or right ventricular function, and/or evaluation of myocardial fibrosis, and evaluation of myocarditis/myocardial inflammation (n = 15, 13.2%). The evaluation for myocarditis or myocardial inflammation was performed in patients who presented with chest pain and elevated cardiac biomarkers, especially when obstructive coronary artery disease was excluded.

Another common indication for CMR was evaluation of ischaemia and myocardial viability (n = 12, 10.5%). These assessments are performed on patients with unexplained LV dysfunction or patients who have suffered from a prior myocardial infarction (MI). Furthermore, another indication for CMR (n = 11, 9.6%) is the evaluation of arrhythmia; this includes assessment of structural heart disease in patients presenting with atrial or ventricular arrhythmias. It was also found that only a small percentage of total referrals (n = 5, 4.4%) represented congenital or structural heart disease. Lastly, approximately 50 (43.9%) referrals were grouped under ‘other’ indications. These include conditions such as chemotherapy-induced cardiomyopathy, heart failure, and chest pain of unknown origin. This further helps to demonstrate the usefulness of CMR as a comprehensive diagnostic tool in rural clinical practice. Table 2 summarises primary clinical indications.

**Table 2:** Primary Clinical Indications for Cardiac MRI A chi-squared goodness-of-fit test was used to assess whether the distribution of clinical indications differed significantly from an equal expected distribution. *Other/mixed indications included complex heart failure presentations, chemotherapy-related cardiomyopathy, chest pain of unclear aetiology, and multifactorial referrals. χ²: chi-squared statistic

Clinical indication	Number of scans (%)	95% confidence interval	χ² statistic	p-value
Cardiomyopathy assessment	21 (18.4%)	12.4-26.5		
Myocarditis assessment	15 (13.2%)	8.1-21.0		
Ischaemic scar evaluation	12 (10.5%)	6.1-17.5		
Arrhythmia-related assessment	11 (9.6%)	5.5-16.4		
Structural heart disease	5 (4.4%)	1.9-9.8		
Other/mixed indications*	50 (43.9%)	35.1-53.0		
Overall distribution			χ² = 67.89	<0.001

The most frequent type of CMR study was cardiac myocardial assessment, which accounts for 53 studies (46.5%; 95% CI: 37.6%-55.6%). The distribution of clinical indications differed significantly from an equal expected distribution (χ² = 67.89, p < 0.001). These scans were used to assess for myocardial scarring, cardiomyopathy, tissue viability, and ventricular function in the study group.

Additionally, MRI cardiac morphology accounted for the second-largest subset of study types, consisting of 50 (43.9%; 95% CI: 35.1%-53.0%) studies. These studies were used to assess anatomical features of structural cardiac defects and the effect of these defects on patient cardiac function. The high number of this type of studies suggests the role of CMR as a primary non-invasive imaging modality to assist in the evaluation of patients with structural heart diseases. In addition, MRI cardiac complex studies represented nine studies (7.9%; 95% CI: 4.2%-14.3%). MRI cardiac complex studies usually require extra imaging sequences to allow a thorough assessment of the patient's cardiac anatomy, resulting in longer scan times compared with other CMR categories. Overall, there is a significant difference between the distribution of CMR study types and what would be an equally distributed pattern (χ² = 122.49; p < 0.001). As part of this evaluation, it was found through Pearson residual analysis that there was a greater frequency than expected for both myocardial assessment and morphology studies while a lower frequency than expected for all combinations of these studies.

Furthermore, few studies have included the combination of two different types of imaging protocols. For example, one MRI cardiac complex study was combined with an MRI head with contrast (n = 1, 0.9%; 95% CI: 0.16%-4.8%), and one magnetic resonance angiography (MRA) aorta whole study was combined with an MRI cardiac morphology study (n = 1, 0.9%; 95% CI: 0.16%-4.8%). These examples demonstrate the flexibility of combining CMR with other imaging protocols in complex clinical cases. Table 3 summarises the distribution of each type of scan.

**Table 3:** Summary of Cardiac MRI Study Types A chi-squared goodness-of-fit test was used to assess whether the observed distribution of CMR study types differed from an equal expected distribution. χ²: chi-squared statistic; CMR: cardiac magnetic resonance imaging; CI: confidence interval

Study description category	Number of scans	Percentage (%)	95% CI	χ² statistic	p-value
MRI cardiac myocardial assessment	53	46.5	37.6-55.6		
MRI cardiac morphology	50	43.9	35.1-53.0		
MRI cardiac complex	9	7.9	4.2-14.3		
Complex + head	1	0.9	0.2-4.8		
Aorta + morphology	1	0.9	0.2-4.8		
Chi-squared goodness-of-fit test				χ² = 122.49	<0.001

The results highlight that the contrast agent of choice for most of these scans was gadolinium-based, accounting for 111 studies (97.4%). However, three (2.6%) studies did not use contrast. Two of these studies were performed without contrast due to the patient being pregnant, and one due to a previous allergy.

Overall, this hospital's quality of imaging was very good. Overall, 92 (80.7%; 95% CI: 72.5%-86.9%) of the total number of scans (n = 114) were categorised as satisfactory quality exams (i.e., optimal or satisfactory). Overall, 92 (80.7%) examinations were considered diagnostically adequate, while 22 (19.3%; 95% CI: 13.1%-27.5%) were classified as suboptimal/technically limited (Table 4). Despite these technical limitations, all 22 examinations remained diagnostically interpretable, and no examination was reported as non-diagnostic. These results suggest that rural hospitals using suitable techniques and personnel may be able to produce both technologically sufficient and clinically useful CMR exams.

**Table 4:** Image Quality Table CI: confidence interval

Image quality	n (%)	95% CI
Good/satisfactory	92 (80.7%)	72.5-86.9
Suboptimal/technically limited	22 (19.3%)	13.1-27.5

The use of CMR to diagnose patients demonstrated a wide range of diagnostic data that resulted in abnormal results in the majority of these tests. For example, the most common findings in these patients included myocardial scar or fibrosis, accounting for 51 (44.7%) MRI studies. Furthermore, alongside ischaemic scars, many non-ischaemic scars were detected, helping to distinguish between coronary and non-coronary causes of myocardial injury.

The second most common diagnosis found by these CMRs is myocarditis or myocardial inflammation, accounting for 24 (21.1%) cases. These scans showed evidence of myocardial oedema, without evidence of ischaemia, indicating the myocardium was damaged due to inflammatory processes. Additionally, 20 (17.5%) scans reported no evidence of structural or tissue abnormalities, highlighting the usefulness of CMR in providing reassurance and high degrees of confidence that no such abnormalities exist.

Moreover, other abnormalities picked up by the CMR included cardiomyopathy or LV systolic dysfunction, accounting for seven (6.1%) cases; LV hypertrophy, accounting for three (3.0%) cases; and mixed or incidental findings, accounting for nine (7.6%) cases. Table 5 summarises the diagnostic findings on CMR.

**Table 5:** Summary of Cardiac MRI Findings Data are shown as number and percentage with corresponding 95% confidence intervals. A chi-squared goodness-of-fit test was used to assess whether the observed distribution of cardiac MRI findings differed from an equal expected distribution. χ²: chi-squared statistic; LV: left ventricular

Cardiac MRI finding category	Number	Percentage (%)	95% confidence interval	χ² statistic	p-value
Myocardial scar/fibrosis	51	44.7	35.9-53.9		
Myocarditis/inflammatory changes	24	21.1	14.6-29.4		
No significant abnormality	20	17.5	11.7-25.6		
Cardiomyopathy/LV dysfunction	7	6.1	3.0-12.1		
LV hypertrophy	3	3.0	0.9-7.5		
Mixed/incidental findings	9	7.6	4.2-14.3		
Overall distribution				χ² = 81.58	<0.001

**Figure 1:**
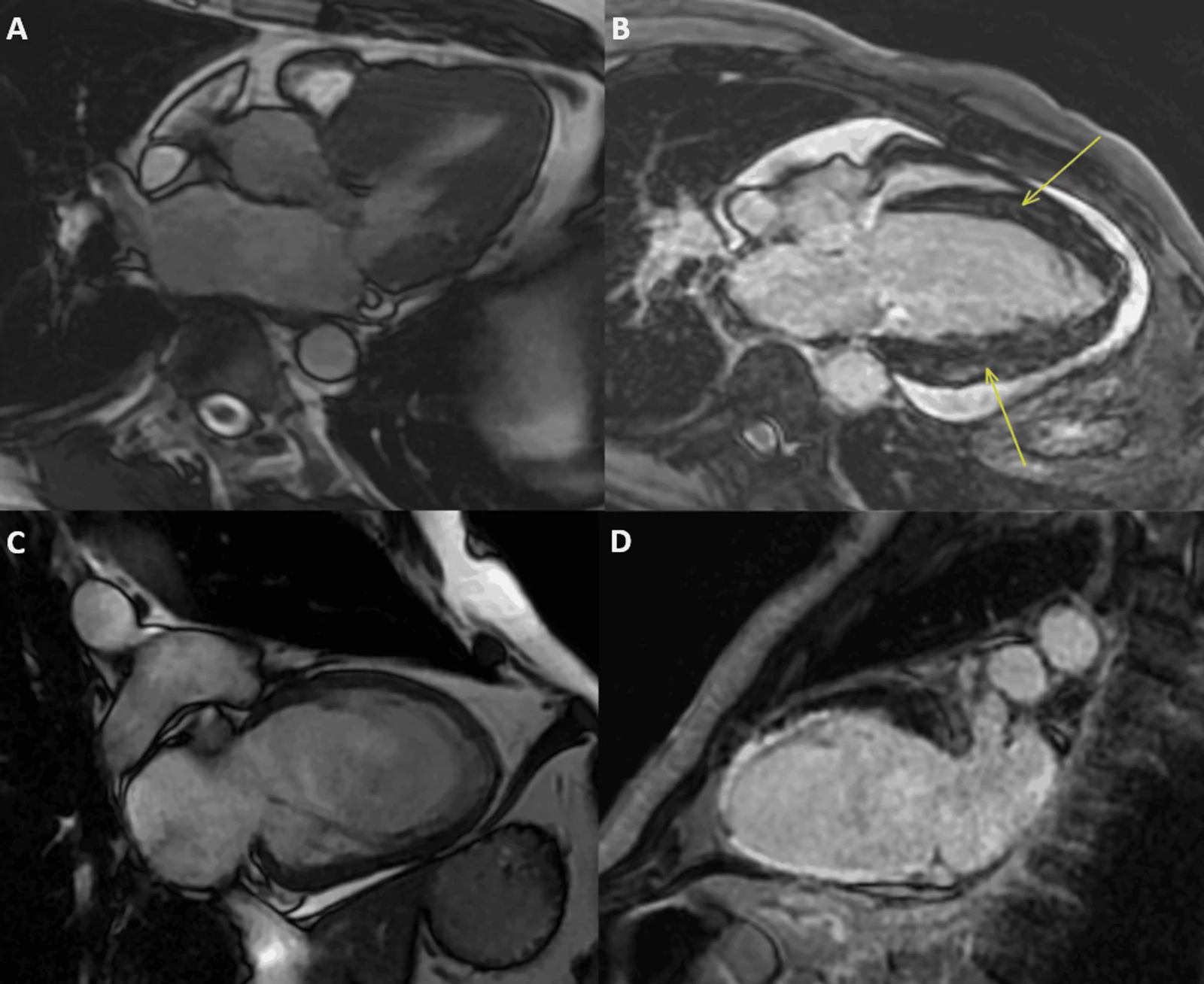
Representative Cardiac Magnetic Resonance Findings From the Study Cohort (A) Hypertrophic obstructive cardiomyopathy: three-chamber cine view demonstrating left ventricular hypertrophy with subaortic stenosis. (B) Acute myocarditis: four-chamber view demonstrating patchy intramyocardial late gadolinium enhancement (arrows), left ventricular dilatation, and pericardial effusion. (C) Dilated cardiomyopathy: two-chamber cine view demonstrating left ventricular enlargement without myocardial hypertrophy. (D) Myocardial infarction: two-chamber late gadolinium enhancement view demonstrating diffuse subendocardial and transmural left ventricular enhancement.

Representative CMR examples showing diagnostic findings identified within the cohort are presented in Figure 1. Figure 1A demonstrates hypertrophic obstructive cardiomyopathy, Figure 1B shows acute myocarditis, Figure 1C demonstrates dilated cardiomyopathy, and Figure 1D shows MI.

CMR demonstrated a clinically relevant impact across the cohort. In 46 (40.4%; 95% CI: 31.8%-49.5%) examinations, CMR confirmed or excluded the suspected diagnosis or clinical concern prompting referral. A new or previously unrecognised diagnosis was identified in 28 (24.6%; 95% CI: 17.6%-33.2%) examinations. In 19 (16.7%; 95% CI: 10.9%-24.6%) examinations, the CMR findings directly influenced subsequent patient management, including treatment, further investigation, or referral. In 21 (18.4%; 95% CI: 12.4%-26.5%) examinations, CMR provided diagnostic reassurance without a documented change in management. These four outcomes were treated as mutually exclusive primary categories, with each examination assigned to the single most clinically relevant outcome (Table 6).

**Table 6:** Impact of Cardiac MRI on Patient Management A chi-squared goodness-of-fit test was used to evaluate whether management outcomes differed from an equal expected distribution. χ²: chi-squared statistic; CI: confidence interval

Management outcome	n (%)	95% CI	χ² statistic	p-value
Diagnostic confirmation/exclusion	46 (40.4%)	31.8-49.5		
New/unexpected diagnosis	28 (24.6%)	17.6-33.2		
Reassurance	21 (18.4%)	12.4-26.5		
Aid patient management	19 (16.7%)	10.9-24.6		
Chi-squared goodness-of-fit test			χ² = 15.89	0.0012

There were some documented minor events with CMR, including intolerance or breath-holding difficulties in six (5.3 %) cases. Nevertheless, there was no documented allergic reaction to the gadolinium-based contrast. Overall, the results indicate that CMR is shown to be safe and tolerable as an imaging tool within rural hospitals.

## Discussion

This retrospective observational study focused on indications, technical performance, and clinical implications of CMR at a rural hospital between 2024 and 2025. The results suggested that CMR was primarily used as a diagnostic tool for evaluating suspected ischaemia, myocarditis, and cardiomyopathies. Although the rural hospital setting presented its own challenges, the CMR studies performed at this hospital were found to have high diagnostic quality and had a significant impact on patients. Furthermore, there had been notable differences in how CMR was utilised at this rural centre compared to other tertiary and metropolitan centres.

Clinical indications compared with published CMR cohorts

In our cohort, cardiomyopathy assessment accounted for 18.4% of CMR referrals, while myocarditis assessment accounted for 13.2% and ischaemic scar evaluation for 10.5%. In comparison, the EuroCMR registry reported suspected coronary artery disease/ischaemia as the most common indication (34.2%), followed by myocarditis/cardiomyopathy (32.2%) and myocardial viability assessment (14.6%) [13,14]. The lower proportion of ischaemia-related referrals and the higher proportion of other or mixed indications (43.9%) in our cohort may reflect differences in referral pathways and local service configuration. However, direct comparison should be interpreted cautiously because the indication categories and healthcare settings differed between cohorts [5,14].

The results of this cohort, compared to other published data, show that the referrals for suspected myocarditis were greater. This is because registry data show that myocarditis generally accounts for a smaller portion of total CMR usage, typically being less than 10% [15]. The greater proportion of myocarditis in this cohort may represent a higher clinical suspicion of inflammatory myocardial disease in patients who present with chest pain, elevated troponin levels, or unobstructive coronary artery disease. The usefulness of CMR in diagnosing myocarditis is well documented, especially after the revised Lake Louise Criteria were released, which improved the sensitivity and specificity for myocarditis [16]. CMR provides an important non-invasive component of the diagnostic assessment of suspected myocarditis, particularly where endomyocardial biopsy is not clinically indicated.

In this cohort, 43.9% of referrals were classified as having other or mixed indications, including complex heart failure presentations, chemotherapy-related cardiomyopathy, chest pain of unclear aetiology, and multifactorial referrals. These findings suggest that there are many different factors affecting the referral practices in rural hospitals. For example, many patients who were referred for CMR in rural hospitals had multiple comorbid conditions and/or a high degree of diagnostic uncertainty when they were referred. Other studies have identified similar trends in reports of service evaluation from non-tertiary centres, where they highlight that CMR can provide comprehensive assessment of multiple cardiac parameters within a single examination, allowing evaluation of different clinical questions, including myocardial structure, ventricular function, and tissue characterisation [17].

Protocol selection and non-stress CMR

Another notable difference between this cohort and published tertiary-centre practice was the absence of pharmacological stress CMR in our cohort. Pharmacological stress CMR is an established non-invasive method for assessing myocardial ischaemia and has demonstrated good diagnostic performance when compared with other approaches to ischaemia assessment [18,19]. In the MR-INFORM trial, a myocardial-perfusion CMR-guided strategy was non-inferior to a fractional flow reserve (FFR)-guided strategy for major adverse cardiac events in patients with stable angina, supporting its role in selected patients undergoing assessment for coronary disease [19]. However, the reasons for the absence of stress CMR in our cohort were not directly assessed and therefore cannot be attributed definitively to service-level limitations. Pharmacological stress CMR has been shown to have high specificity and sensitivity and is demonstrated to be a valuable tool in assessing a specific group of patients (Table 7) [8,19].

**Table 7:** Comparison of CMR Utilisation Between the Present Rural Cohort and Published Evidence CMR: cardiac magnetic resonance imaging

Characteristic	Present rural cohort	Published evidence
Pharmacological stress CMR	0% (0/114)	Established modality for assessment of myocardial ischaemia [18,19]
CMR myocardial perfusion	Not performed	Demonstrated diagnostic/clinical utility in selected patients [18,19]

The absence of pharmacological stress CMR in this cohort may reflect local service configuration or referral pathways; however, the reasons for this were not directly assessed in the present study. This is because pharmacological stress CMR is associated with higher resource utilisation, in terms of expertise, equipment, and protocols, and therefore may be difficult to consistently maintain in rural hospital services. However, pharmacological stress CMR has higher sensitivity and specificity. Where pharmacological stress CMR is not available, alternative assessment of myocardial ischaemia may include stress echocardiography or nuclear myocardial perfusion imaging, depending on local availability and clinical indication.

Usefulness of contrast and diagnostic yield

Almost every examination performed in this study utilised a gadolinium-based contrast agent, which plays a key role in helping to characterise myocardial tissue. Furthermore, large registry studies demonstrate that LGE is used to provide important diagnostic and prognostic information for a wide range of conditions such as cardiomyopathies, myocarditis, and MI with non-obstructive coronary arteries [6,20,21]. Additionally, the high incidence of myocardial scar or fibrosis (Figure 1) within this cohort mirrors that reported in large CMR registries [13]. A substantial proportion of examinations demonstrated clinically relevant abnormalities, with myocardial scar or fibrosis being the most frequently reported finding (44.7%). Differences in referral patterns and patient selection may contribute to the observed distribution of diagnostic findings [13].

Quality of images and technical feasibility of performing CMR in a rural setting

In this cohort, 19.3% of examinations were classified as suboptimal or technically limited; however, all remained diagnostically interpretable and no examination was considered non-diagnostic. Previous studies have reported non-diagnostic examinations in approximately 3%-10% of cases, although direct comparison is limited by differences in definitions of image quality and diagnostic adequacy [22,23]. The consistent availability of high-quality images in this rural cohort shows that, when standardised protocols and well-trained staff are available, CMR can be successfully performed outside tertiary centres.

Clinical management implications compared to published data

CMR was reported to have a clinical impact in this cohort, including providing diagnostic clarification, identifying previously unrecognised pathology, and influencing documented management decisions. For example, it helped to offer diagnostic clarity, find a new pathology, and affect the patient’s overall management. Furthermore, other tertiary centres reported that in 30%-60% of the cases, CMR changed patient management depending on the study's referral criteria and methodology [17,24]. The clinical implications found in this cohort may be due to different factors, such as a highly selected population, substantial diagnostic uncertainty before CMR, and the ability of CMR to assess multiple parameters in a single study. This makes CMR valuable in rural areas, where access to repeated tests is limited.

Significance for rural health delivery

Rural populations often face significant barriers to obtaining more specialised diagnostic services. The results of this study indicate that when CMR is available, it can be safely and effectively used to guide clinical management and improve overall patient care. However, limited training, education, expertise, and restricted access to CMR services contribute to a significant gap in rural healthcare provision. Addressing this gap may involve increasing workforce capacity, allocating resources appropriately, upgrading equipment and software, expanding training and education opportunities, and improving clinicians’ awareness of guideline-supported CMR indications. The development of regional reporting systems could further support service consistency. In addition, tele-radiology services involving subspecialists may offer valuable support in rural settings with constrained staffing.

Limitations

This study has several limitations. First, the sample size was relatively small (n = 114), and the observation period was limited to one year, which may limit the generalisability of the findings and the ability to assess longer-term trends in CMR utilisation. The study was conducted at a single rural hospital without a comparator urban or tertiary centre, limiting direct comparison between rural and urban CMR practice. Potential selection and referral bias may also have influenced the observed indications and diagnostic findings, as the cohort consisted of patients selected for CMR through local referral pathways. Furthermore, clinical impact was retrospectively determined from documented reports and clinical information rather than prospectively assessed patient outcomes, which may have resulted in under- or over-estimation of management influence. The categorisation of clinical indications, diagnostic findings, and clinical-impact outcomes may also have introduced misclassification. Finally, reliance on finalised radiology reports as the primary source of diagnostic and clinical-impact information may have introduced reporting and interpretation bias, as the level of detail and terminology used in reports may have varied between examinations and reporting clinicians.

Future directions

Future research should incorporate predefined measures of clinical impact and assess referral-to-scan and scan-to-treatment intervals, downstream investigations, treatment changes, healthcare utilisation and associated costs, and patient outcomes. Comparative studies involving rural and urban/tertiary services would also help to identify differences in CMR utilisation, service delivery, and clinical impact.

## Conclusions

In summary, CMR was frequently used for the assessment of cardiomyopathy, myocarditis, and ischaemic disease in this rural hospital cohort. Most examinations were diagnostically adequate, and CMR findings were documented as contributing to diagnostic clarification, identification of previously unrecognised abnormalities, or management decisions in a proportion of examinations. However, the absence of a comparator centre and prospective patient-outcome data limits conclusions regarding effectiveness or broader impact on healthcare delivery. These findings provide a description of CMR utilisation and documented clinical impact within this rural service and may inform future comparative studies of rural and tertiary CMR provision.
